# Research note: Age and time of day influence broiler use of platform enrichments

**DOI:** 10.1016/j.psj.2026.106720

**Published:** 2026-02-27

**Authors:** Shawna L. Weimer, Michael T. Kidd, Mitchell Vaught, Seth Holeyfield, Benjamin Angel, Pablo Ruidiaz Escovar, Rovin Sanjur Caballero, Karen Pitty Rivera, Elle Johnston, Seong W. Kang, Rosie H. Whittle

**Affiliations:** Department of Poultry Science, University of Arkansas, 1260 W Maple St, Fayetteville, AR 72701, United States

**Keywords:** Broiler, Welfare, Platform enrichment, Scotoperiod, Wall-hugging behavior

## Abstract

Environmental enrichments have the potential to improve broiler chicken welfare. Platforms, when designed and placed effectively, can improve spatial complexity and encourage positive natural behavior. This study evaluated the spatial and temporal patterns of platform use in broilers housed with sidewall-mounted platforms compared to unenriched controls. A total of 352 male broilers were randomly assigned to one of two environmental enrichment treatments: enriched pens with white plastic slatted platforms placed along both sidewalls (Platform, n=6 pens) and unenriched controls with no platforms (Control, n=2 pens). Overhead video recordings were captured, and behavioral observations of side occupancy were conducted using instantaneous scan sampling at 15-min intervals for 24 h on d 16, 23, 30, 37, 44, and 50 of age. Birds in platform pens had greater side occupancy than controls at all ages and lighting conditions, except during the photoperiod on d 50. The most pronounced differences occurred at younger ages during the scotoperiod, with over 80% of the birds on the sides of platform pens during the scotoperiod on d 16 and 23, compared to less than 40% in control pens (P<0.0001). Less pronounced platform use patterns were observed during the photoperiod. The most significant difference in side occupancy during the photoperiod was on d 30, where 27% more birds were on the sides in platform pens compared to controls (P<0.0001). Taken together, these results suggest that even in the absence of platforms, broilers showed a preference for the sides of the pen (thigmotaxic behavior) during the scotoperiod. The presence of platforms augmented this behavior, particularly during the scotoperiod and at younger to mid-growth ages.

## Introduction

Environmental enrichment is increasingly recognized as an on-farm component to improving the welfare of broiler chickens by promoting comfort and play behaviors, physical activity, and a positive affective state (Jacobs et al., 2023). Among enrichment strategies, elevated platforms have shown promise to improve welfare by increasing spatial complexity and encouraging locomotion in broilers. Structural enrichments such as platforms and perches have been evaluated to improve welfare by increasing activity and reducing lameness (Riber et al., 2018; Vasdal et al., 2019). However, environmental enrichments are only effective if they elicit meaningful behavioral changes that improve welfare (Newberry, 1995) and are consistently used as birds age (Riber et al., 2018). Their effectiveness depends not only on the type of enrichment but also on its design, placement, and attractiveness to the birds.

Platform environmental enrichment designs vary across studies in terms of material, height above ground, dimensions, shape, and access ramp dimensions (Riber et al., 2018), but location within the home environment also affects use. The relationship between space use and resource availability is well established. More available space leads to greater enrichment use, with smaller groups of broilers showing stronger spatial preferences and higher engagement with enrichments than larger groups (Leone and Estévez, 2008; Malchow and Schrader, 2021; Vas et al., 2023). Platform use tends to decline with age, not necessarily due to reduced interest, but because birds grow larger while the platform space remains fixed, limiting occupancy (Malchow & Schrader, 2021; Lopez et al., 2022).

Optimizing enrichment use involves understanding the natural spatial preferences of broilers. Thigmotaxis, or wall-hugging behavior, reflects a tendency to stay near walls, possibly as an anti-predator strategy or to avoid disturbances during rest (Buijs et al., 2010). Broilers prefer using resources in peripheral space rather than in open space (Newberry, 1999), and proximity to walls has been shown to reduce social stress and competition near feed and water lines (Arnould and Faure, 2003). Broilers are known to cluster near walls at higher stocking densities, indicating a density-dependent spatial preference (Arnould and Faure, 2003; Buijs et al., 2010). Given these spatial preferences, the present study aimed to investigate broiler platform enrichment use by positioning platforms along the periphery of the home environment.

Platforms offer broilers opportunities for exploration, rest, and escape from the litter (Riber et al., 2018; Malchow & Schrader, 2021). Yet little is known about how their use changes throughout the day and across developmental stages. The objective of this study was to evaluate the use of platforms by broiler chickens during 24-h periods across six ages from week two through week seven of age. We further examined temporal patterns of platform use between the photoperiod and scotoperiod.

## Materials & methods

### Animals and housing

The study was conducted at the University of Arkansas Applied Broiler Research Unit, and the University of Arkansas Division of Agriculture Animal Care and Use Committee approved all methods and procedures. Day-of-hatch male broiler chickens (n=352) were randomly assigned to eight floor pens (1.83 m x 2.44 m, n=44/pen) with clean pine shavings litter and ad libitum access to feed and water. Housing conditions were maintained according to the Cobb-Vantress broiler management guide (Cobb-Vantress, 2021), with a photoperiod of 16 h and a scotoperiod of 8 h. Pens were randomly assigned to one of two environment enrichment treatments: white plastic slatted platforms placed along both sidewalls (n=6 pens) and unenriched controls, with (no platforms, n=2 pens). The platforms were 2.44 m long x 25.4 cm wide x 12.7 cm tall, attached to a vinyl ramp at a 39-degree angle at the bottom and 51-degree angle at the top.

### Behavioral observation

**Fig. 1:**
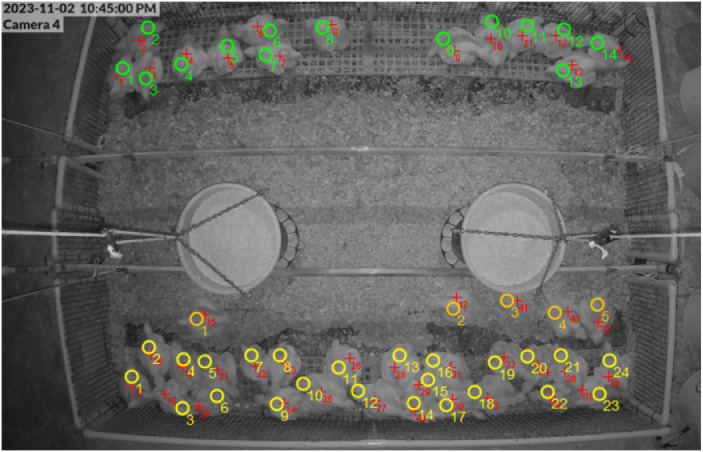
Overhead image of a platform pen during the scotoperiod on d 23. Colored circles and numerical labels indicate the total number of birds (red), and positions within the pen: east platform (green), west platform (yellow), and litter area (orange) of the pen. Images were measured using ImageJ.

Overhead video recordings were captured using eight ceiling-mounted closed-circuit television (CCTV) cameras (G4 bullet, Ubiquiti, New York, NY, USA), positioned at the center of each pen to provide a full aerial view. Continuous 24-h video recordings were collected on d 16, 23, 30, 37, 44, and 50 of age. Behavioral observations were conducted using instantaneous scan sampling at 15-min intervals for all the birds in each pen (96 scans/pen/day). Screenshot images were extracted at each interval and analyzed using ImageJ software (National Institute of Health, Bethesda, Maryland, USA; Schneider et al., 2012). The number of birds located in the litter area and on the platforms was recorded using the count tool (Fig. 1). A bird was counted as being on a platform if more than 50% of its body was located on the platform surface. For control pens, a transparency overlay with a marker tracing the pixel dimensions of the platform was used to record the number of birds that occupied the equivalent space in the pen. The converted platform dimensions were calculated by counting the same number of plastic mesh squares visible along the pen sidewalls from a digital image of a platform pen. These mesh squares served as a reference grid to calibrate the platform dimensions in ImageJ. For each scan, the number of birds located on the platforms, or equivalent areas in control pens, was divided by the total number of birds visible in the pen and multiplied by 100 to obtain the percentage of birds on the sides. For statistical analysis, scan data were aggregated into average proportions of birds on the litter and platforms within 4-h time blocks to reduce short-term variability and facilitate comparisons across ages and lighting conditions (photoperiod and scotoperiod).

### Statistical analysis

To determine the difference in the percentage of birds located in the litter versus on the platforms, data were analyzed in R. A linear mixed-effects model (“lme4” package) was fitted with fixed effects of treatment (platform vs. control), d of age (16, 23, 30, 37, 44, and 50), and photoperiod (photoperiod vs. scotoperiod) and their two- and three-way interactions. Data did not meet the assumptions of normality, and a square root transformation was applied before analysis. Pen and 4-h time block were included as random effects to account for repeated measures and pen-level variation. Post hoc pairwise comparisons of estimated marginal means were conducted using the “emmeans” package with Holm’s adjustment for multiple comparisons. Estimated means were back transformed for interpretation and are reported as estimated means ± the SE. Statistical significance was set at P≤0.05.

## Results & discussion

In this study, we quantified the spatial and temporal patterns of platform use by broiler chickens from week two through seven of age, using occupancy of the pen sides as a proxy for engagement with platform enrichments. The percentage of birds on the sides of the pen was measured in both platform-enriched and control pens during the photoperiod (lights on) and scotoperiod (lights off). Overall, platform enrichments increased side occupancy and thereby influenced bird distribution, with effects varying by age and lighting condition.

**Fig. 2:**
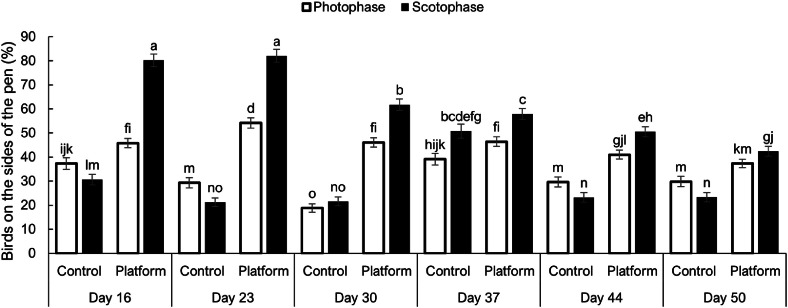
Percentage of broiler chickens observed on the sides of Platform and Control pens during the photoperiod and scotoperiod of 24 h at 16, 23, 30, 37, 44, and 50 d of age.

Platform enrichments most strongly increased wall-hugging behavior during the scotoperiod at younger ages (P<0.0001). At 16 and 23 d of age, more than 80% of birds in platform pens were located along the pen sides during the scotoperiod, compared with less than 40% in control pens (P<0.0001). The greatest difference occurred on d 16, with 34.4% more birds on the sides in platform pens. This difference gradually decreased with age, with treatment differences of 27.8%, 15.7%, 11.5%, 9.5%, and 5.1% at 23, 30, 37, 44, and 50 d of age, respectively (Fig. 2). Age-related decreases in platform use are consistent with previous reports that enrichment use declines as the birds grow and the available space per bird on the enrichment becomes more limited (Malchow & Schrader, 2021; Lopez et al., 2022).

Wall-hugging behavior (thigmotaxis) reflects a tendency to stay near walls, possibly as an anti-predator strategy or to avoid disturbances during rest (Buijs et al., 2010). In the present study, thigmotaxic behavior was clearly influenced by age and photoperiod (Fig. 2). The increased side occupancy in platform pens, especially during the scotoperiod, suggests that platforms not only provide a physical resource but also improve the ability of the birds to use preferred peripheral space, reinforcing their natural spatial preferences.

During the photoperiod, patterns of platform use were less pronounced than during the scotoperiod. Interestingly, birds in control pens occupied the pen sides more during the photoperiod than the scotoperiod on d 16 and 23 (P<0.0001), and on d 16 there was no difference between platform and control pens (Fig. 2). The greatest treatment difference during the photoperiod occurred on d 30, where 27.3% more birds were on the sides in platform pens than in control pens (P<0.0001). At older ages, bird distribution in control pens became more uniform across age and lighting periods, with side occupancy remaining relatively low and stable, ranging from 29.8% to 23.2% on d 44 and 50. For d 37 and 50, there were no differences in side occupancy between treatments during the photoperiod, indicating a reduced effect of platforms on lateral space use during the light period.

Previous work has reported both greater platform use during the photoperiod (Norring et al., 2016; Lopez et al., 2022) and greater use during the scotoperiod (Malchow et al., 2019, 2025), suggesting temporal patterns of use may depend on enrichment design, placement, and flock management. In the current study, the strongest treatment effects were observed at night, when the birds likely used the platforms as preferred roosting sites. The relationship between platform location and natural thigmotactic tendencies may have contributed to the high nighttime occupancy in peripheral areas, especially at younger ages when more space was available on the platforms.

Taken together, these results suggest that even in the absence of platforms, broilers showed a preference for the sides of the pen during the scotoperiod, though to a much lesser extent than in platform pens. The presence of sidewall-mounted platforms fostered this wall-hugging behavior, particularly during the scotoperiod from early to mid-growth ages. These results highlight the importance of both enrichment design and placement, as well as the timing of enrichment use, in maximizing welfare benefits. Providing elevated resting areas along the periphery supports natural roosting behavior and offers birds opportunities to rest away from high-activity areas, especially at younger ages. Future studies should further explore the motivational mechanisms underlying these spatial preferences and evaluate how enrichment strategies can be optimized for different stages of broiler development under commercial conditions.

## Funding

This project was funded by the Center for Food Animal Wellbeing.

## CRediT authorship contribution statement

**Shawna L. Weimer:** Writing – review & editing, Writing – original draft, Visualization, Validation, Supervision, Software, Resources, Project administration, Methodology, Investigation, Funding acquisition, Conceptualization. **Michael T. Kidd:** Writing – review & editing, Supervision, Resources, Project administration. **Mitchell Vaught:** Investigation, Data curation. **Seth Holeyfield:** Investigation, Data curation. **Benjamin Angel:** Investigation, Data curation. **Pablo Ruidiaz Escovar:** Data curation. **Rovin Sanjur Caballero:** Data curation. **Karen Pitty Rivera:** Data curation. **Elle Johnston:** Data curation. **Seong W. Kang:** Writing – review & editing, Writing – original draft. **Rosie H. Whittle:** Writing – review & editing, Validation, Formal analysis.

## Disclosures

The authors declare no conflicts of interest.
